# Lineage-Specific Rewiring of Core Pathways Predating Innovation of Legume Nodules Shapes Symbiotic Efficiency

**DOI:** 10.1128/mSystems.01299-20

**Published:** 2021-04-13

**Authors:** Wen-Jing Cui, Biliang Zhang, Ran Zhao, Li-Xue Liu, Jian Jiao, Ziding Zhang, Chang-Fu Tian

**Affiliations:** a State Key Laboratory of Agrobiotechnology and College of Biological Sciences, China Agricultural University, Beijing, China; b MOA Key Laboratory of Soil Microbiology and Rhizobium Research Center, China Agricultural University, Beijing, China; University of Hawaii at Manoa

**Keywords:** adaptation, immunity, mutualism, pangenome, legume

## Abstract

The interkingdom coevolution innovated the rhizobium-legume symbiosis. The application of this nitrogen-fixing system in sustainable agriculture is usually impeded by incompatible interactions between partners. However, the progressive evolution of rhizobium-legume compatibility remains elusive. In this work, deletions of *rhcV* encoding a structural component of the type three secretion system allow related *Sinorhizobium* strains to nodulate a previously incompatible soybean cultivar (Glycine max). These *rhcV* mutants show low to medium to high symbiotic efficiency on the same cultivated soybean while being indistinguishable on wild soybean plants (Glycine soja). The dual pantranscriptomics reveals nodule-specific activation of core symbiosis genes of *Sinorhizobium* and *Glycine* genes associated with genome duplication events along the chronogram. Unexpectedly, symbiotic efficiency is in line with lineage-dependent transcriptional profiles of core pathways which predate the diversification of Fabaceae and *Sinorhizobium.* This is supported by further physiological and biochemical experiments. Particularly, low-efficiency nodules show disordered antioxidant activity and low-energy status, which restrict nitrogen fixation activity. Collectively, the ancient core pathways play a crucial role in optimizing the function of later-evolved mutualistic arsenals in the rhizobium-legume coevolution.

**IMPORTANCE** Significant roles of complex extracellular microbiota in environmental adaptation of eukaryotes in ever-changing circumstances have been revealed. Given the intracellular infection ability, facultative endosymbionts can be considered pioneers within complex extracellular microbiota and are ideal organisms for understanding the early stage of interkingdom adaptation. This work reveals that the later innovation of key symbiotic arsenals and the lineage-specific network rewiring in ancient core pathways, predating the divergence of legumes and rhizobia, underline the progressive evolution of rhizobium-legume compatibility. This insight not only is significant for improving the application benefits of rhizobial inoculants in sustainable agriculture but also advances our general understanding of the interkingdom coevolution which is theoretically explored by all host-microbiota interactions.

## INTRODUCTION

Despite significant roles of complex extracellular microbiota in environmental adaptation of eukaryotes in ever-changing circumstances (1, 2), rare microorganisms are indispensable for eukaryotes and vertically transmitted from parent to offspring (3, 4). Facultative intracellular microsymbionts are not strictly associated with their hosts but may confer adaptive benefits to hosts under particular conditions (5). Given the intracellular infection ability, facultative endosymbionts can be considered pioneers within complex extracellular microbiota and are ideal organisms for understanding the early stage of interkingdom adaptation.

Among diverse soil bacteria, more than 200 rhizobial species belonging to 18 genera of proteobacteria can induce and intracellularly infect nodules on diverse legumes (6). This is largely explained by the horizontal transfer of nodulation and nitrogen fixation genes among soil bacteria and subsequent recruitment of lineage-specific accessory genes (7–10). Despite the great diversity of rhizobia in the rhizosphere (11–13), the majority of nodules were individually infected by a single clone, a phenomenon called competitive nodulation (14–17). In most cases, legume roots exude flavonoids which specifically interact with a rhizobial transcriptional factor, NodD, to activate the expression of nodulation genes directing the synthesis of Nod factors (18). Specific perception of Nod factors by host receptors activates cascades of signal transduction and subsequent initiation of root hair curling, infection thread formation, and nodule organogenesis (19). The uptake of rhizobia into nodule cells leads to formation of symbiosome, consisting of differentiated rhizobial cell(s) called bacteroids which are surrounded by a host-derived membrane (20). Mature bacteroids are nongrowing and can reduce dinitrogen into ammonium that is exchanged for carbon and other nutrients from host cells (21–23), showing organelle-like features (23, 24). Great variations in nodulation and nitrogen fixation efficiency are commonly observed in agriculture practices (25). However, very few efforts have been undertaken to understand mechanisms underlying these variations (26–30).

*Sinorhizobium* strains are characterized by their multipartite genomes and are intensively studied as model facultative microsymbionts in mutualistic interactions and adaptive evolution of pangenome (23, 31–34). Diverse related *Sinorhizobium* strains have been isolated from soybean nodules in fields of alkaline-saline soils (11, 35, 36) and harbor an open pangenome (10). These strains exhibit notable variations in symbiotic compatibility (29), despite their conserved composition of Nod factors (37). We have recently discovered fast adaptive evolution of narrow-host-range strains into microsymbionts of previously incompatible soybean cultivars (29), and this process is efficiently mediated by parallel transpositions of insertion sequences into the T3SS (type three secretion system) gene cluster composed of genes encoding structural components of the T3SS (e.g., *rhcV*), the effector protein NopP, and the positive transcriptional regulator TtsI (29). Despite their improved nodulation ability, these newly evolved facultative microsymbionts differ in their symbiotic efficiency (29). This highlights a progressive evolution of *Sinorhizobium* compatibility with soybeans, though mechanisms underlying their variation of symbiotic efficiency remain unknown.

To get more evolutionary insights into this progressive interkingdom adaptation phenomenon, comparative transcriptomic analyses of symbiotic partners in soybean nodules were performed in a pangenome context, i.e., dual pantranscriptomics. To this end, we constructed *rhcV* mutants of five related *Sinorhizobium* strains including Sinorhizobium fredii CCBAU45436 (SF4), CCBAU25509 (SF2), and CCBAU83666 (SF8), *Sinorhizobium* sp. III CCBAU05631 (SS1), and Sinorhizobium sojae CCBAU05684 (SJ4). These *rhcV* mutants exhibited contrasting symbiotic performance on a test soybean cultivar (Glycine max cv. JD17) while being equally effective on a wild soybean accession (Glycine soja WSD). The dual transcriptome sequencing (RNA-seq) study in a pangenome context was used to analyze transcriptional profiles of core and accessory genes from both rhizobia and host cells in nodule samples. Active symbiotic roles of *Sinorhizobium* core symbiosis genes and host duplicated genes associated with key evolutionary events in the Fabaceae family were revealed. The symbiotic efficiency was, however, associated with lineage-specific rewiring patterns of core pathways predating the diversification of Fabaceae or *Sinorhizobium*. Core processes associated with these transcriptional profiles were further verified by physiological and biochemical experiments. Novel insights were obtained for the progressive evolution of symbiotic compatibility between legume hosts and their facultative microsymbionts, particularly highlighting a previously unknown mechanism mediated by lineage-specific rewiring of ancient core pathways.

## RESULTS AND DISCUSSION

### Bypassing standing variation of key symbiotic arsenals led to improved compatibility with lineage-dependent symbiotic efficiency.

**FIG 1:**
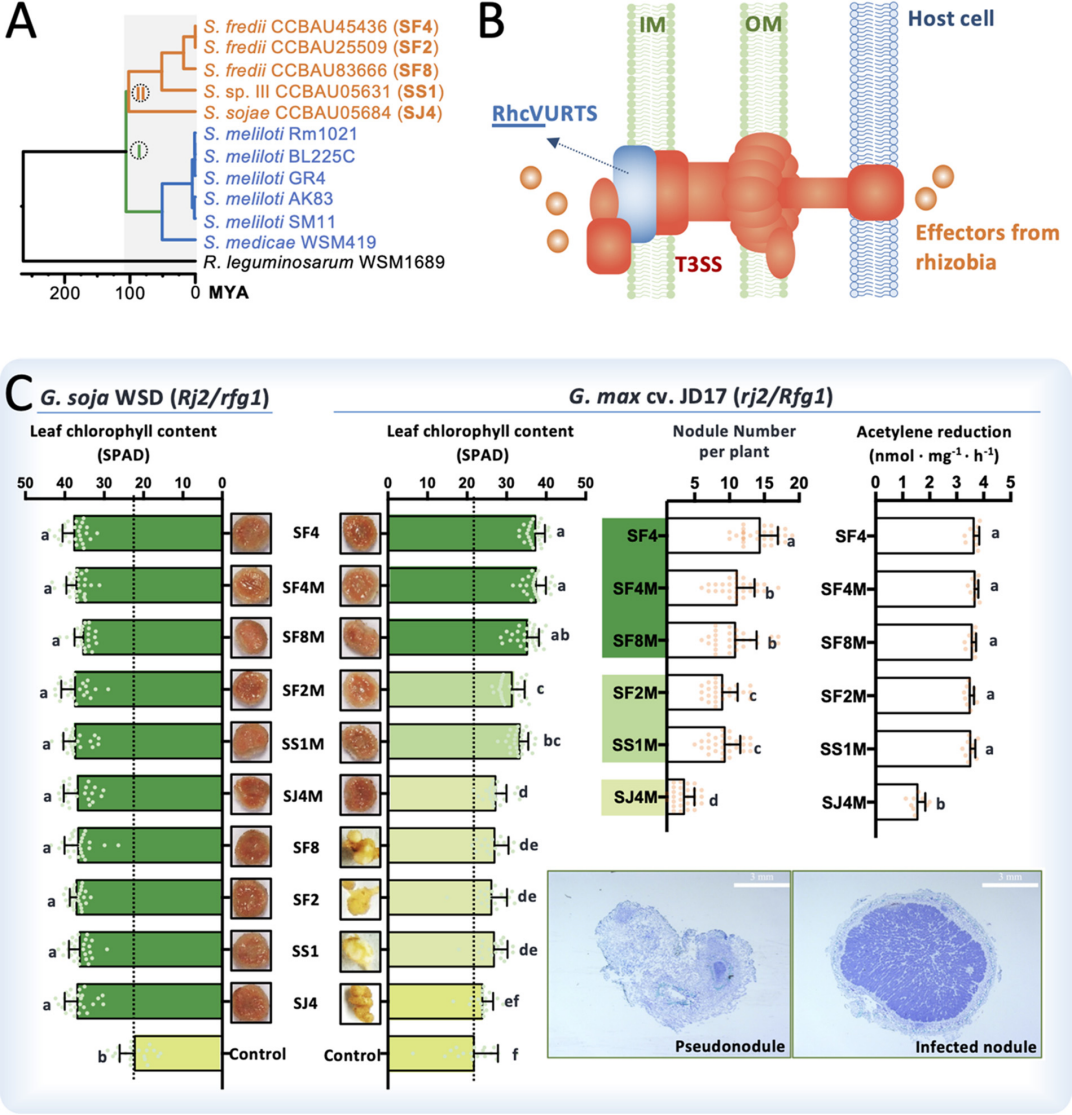
Lineage-specific symbiotic performance of related *Sinorhizobium* strains. (A) A chronogram based on four core genes of strictly vertical evolutionary history. *Sinorhizobium* strains associated with soybeans belong to cluster II. MYA, million years ago. (B) The cluster II strains have a conserved type three secretion system (T3SS). IM, inner membrane; OM, outer membrane. (C) Symbiotic performance of cluster II strains and their mutants lacking *rhcV* (SF4M, SF8M, SS1M, SF2M, and SJ4M), which encodes a structural component of T3SS. Error bars represent SD. Significant differences between means are indicated by different letters based on ANOVA followed by Tukey’s test (alpha = 0.05). Leaf chlorophyll content and nodule numbers were summarized from 18 to 30 scored plants from multiple independent experiments. Acetylene reduction activity was determined using nine plants from three independent experiments. Effective soybean nodules (halves) or pseudonodules/bumps and their representative thin sections (left, a section from pseudonodules induced by SF8; right, a section from effective nodules infected by SF4) are shown.

Independent studies proposed a similar divergence time for *Sinorhizobium-Rhizobium*, as late as 203 million years ago (MYA) (38) or 201 MYA (39, 40), which is much earlier than 60 MYA, when legume (Fabaceae) evolved (41). With 203 to 336 MYA as a calibration point for the *Sinorhizobium-Rhizobium* split (38), the divergence between *Sinorhizobium* strains isolated from soybean nodules and those from *Medicago* nodules was estimated to take place 106 MYA (Fig. 1A), based on four housekeeping genes of strictly vertical evolutionary history in rhizobia (10, 42). A key difference between *Glycine* and *Medicago* microsymbionts is the presence of a functional T3SS in the former strains (Fig. 1B). In this work, the *rhcV* gene encoding an essential structural component of T3SS (43) (Fig. 1B) was deleted in a broad-host-range strain, SF4, and four narrow-host-range strains, SF2, SF8, SS1, and SJ4 (29, 44), resulting in SF4M, SF2M, SF8M, SS1M, and SJ4M, respectively (see Table S1 in the supplemental material). These *rhcV* mutants and their wild-type strains are all compatible with *G. soja* WSD and indistinguishable in symbiotic performance regarding leaf chlorophyll content, nodule number, and nitrogenase activity (Fig. 1C and Table S2). SF4 and five *rhcV* mutants formed nitrogen-fixing nodules on *G. max* cv. JD17 whereas SF8, SF2, SS1, and SJ4 induced pseudonodules on the same host (Fig. 1C). These phenotypes of *rhcV* mutants are similar to those of evolved compatible clones carrying insertion mutation in genes encoding T3SS structural components, TtsI or NopP, as reported previously (29). NopP of *Sinorhizobium* can be translocated into the host cell in a T3SS-dependent manner and can be phosphorylated by host kinases (45, 46). Variations in NopP of *Sinorhizobium* account for the restricted nodulation on *rj2/Rfg1* soybeans but are not required for nodulation on *Rj2/rfg1* soybeans (29, 47, 48). In this work, the Rj2/Rfg1 allelic genotypes of *G. max* cv. JD17 and *G. soja* WSD were determined as *rj2/Rfg1* and *Rj2/rfg1*, respectively, thus supporting previous studies (29, 47). Although it remains unknown how NopP is recognized by different Rj2/Rfg1 soybeans (30, 47), the deletion of *rhcV* in five related *Sinorhizobium* strains allows bypassing the standing NopP variation.

10.1128/mSystems.01299-20.4 TABLE S1 Strains, plasmids, and primers used in this study. Download Table S1, XLSX file, 0.01 MB. Copyright © 2021 Cui et al. 2021 Cui et al. https://creativecommons.org/licenses/by/4.0/ This content is distributed under the terms of the Creative Commons Attribution 4.0 International license.

10.1128/mSystems.01299-20.5 TABLE S2 Symbiotic performance of *Sinorhizobium* strains on *G. max* cv. JD17 and *G. soja* WSD. Download Table S2, XLSX file, 0.01 MB. Copyright © 2021 Cui et al. 2021 Cui et al. https://creativecommons.org/licenses/by/4.0/ This content is distributed under the terms of the Creative Commons Attribution 4.0 International license.

Notably, these *rhcV* mutants are not equally efficient on *G. max* cv. JD17 (Fig. 1C and Table S2). SJ4M showed the lowest nitrogenase activity as measured by acetylene reduction ability (analysis of variance [ANOVA] followed by Tukey’s test, alpha = 0.05). Nodulation ability sequentially increased in the order from SJ4M, SS1M-SF2M, SF8M-SF4M, to SF4, and significant variations in chlorophyll content of plants were also observed among treatments (ANOVA followed by Tukey’s test, alpha = 0.05). Collectively, these six strains can roughly be assigned into the high- (SF4, SF4M, and SF8M), medium- (SF2M and SS1M), and low-efficiency (SJ4M) groups (Fig. 1C). Therefore, conserved composition of the key symbiotic signal (Nod factor) of these related strains (37) and bypassing NopP variation are not enough to confer high-efficiency symbiotic performance on SF2M, SS1M, and SJ4M compared to SF8M and SF4M. Nodulation and nitrogen fixation genes are horizontally transferable and contribute to the great diversity of rhizobial germplasms (7, 8). An ongoing experimental evolution of the plant pathogen Ralstonia solanacearum into legume symbionts after receiving a symbiosis island has succeeded in obtaining clones with improved intracellular symbiosis with the Mimosa pudica legume, though no nitrogen-fixing clones have evolved yet (9, 49), possibly due to the limitation of gene content in this single ancestor strain. In this work, the newly generated symbiotic couples (*rhcV* mutants and *G. max* cv. JD17) with contrasting symbiotic performance may represent ideal materials for further investigation of the progressive evolution of interkingdom compatibility in a pangenome scenario.

### Dual pantranscriptomes of nodules and T3SS-dependent common adaptation pathways.

The dual RNA-seq technique allows simultaneous investigation of interacting bacterium-host partners (50). Here, we extend its application in rhizobium-legume interactions in a pangenome context, i.e., dual pantranscriptomics. Dual RNA-seq was performed for 32 nodule samples from *G. max* cv. JD17 (*G. max* thereafter) and *G. soja* WSD (*G. soja* thereafter) plants inoculated with either wild type or *rhcV* mutants (Tables S3 to S5). Differentially expressed genes (DEGs) in nodules (|Log2R| > 1, adjusted *P* < 0.05) were determined between treatments of individual *rhcV* mutants and SF4 (Fig. S1). Both symbiotic performance and transcriptomes of the SF4M-*G. soja* couple are indistinguishable from the SF4-*G. soja* couple (7 and 0 DEGs in rhizobia and host, respectively). In contrast, the SF4M-*G. max* couple exhibits significant changes in transcriptome compared to SF4-*G. max* (694 and 3,644 DEGs), though no differences in their nitrogenase activity and leaf chlorophyll content were observed (Fig. 1C). In contrast to SF4M, the other test *rhcV* mutants induce various strain- and species-dependent transcriptional changes in *G. soja* nodules compared to either SF4 or their wild-type strains (Fig. S1 and S2), but rare DEGs are shared between multiple treatments. These *rhcV* mutants induce more DEGs in *G. max* than in *G. soja* (Fig. S1), in parallel with their significant difference in symbiotic performance on *G. max* while being indistinguishable on *G. soja* (Fig. 1C and Table S2).

10.1128/mSystems.01299-20.1 FIG S1 Venn plot of differentially expressed genes revealed by dual RNA-seq. All samples from *G. max* cv. JD17 nodules (A) or *G. soja* WSD nodules (B) were compared to the treatment of SF4. No host DEGs were found in WSD nodules induced by SF4 and SF4M. Download FIG S1, TIF file, 1.4 MB. Copyright © 2021 Cui et al. 2021 Cui et al. https://creativecommons.org/licenses/by/4.0/ This content is distributed under the terms of the Creative Commons Attribution 4.0 International license.

10.1128/mSystems.01299-20.2 FIG S2 Venn plot of differentially expressed genes revealed by dual RNA-seq in *G. soja* WSD nodules. All samples from *G. soja* WSD nodules were compared to the individual treatments using corresponding wild-type strains. No host DEGs were found in WSD nodules induced by SF4 and SF4M. Download FIG S2, TIF file, 0.5 MB. Copyright © 2021 Cui et al. 2021 Cui et al. https://creativecommons.org/licenses/by/4.0/ This content is distributed under the terms of the Creative Commons Attribution 4.0 International license.

10.1128/mSystems.01299-20.6 TABLE S3 Basic information on plant expression analysis within *G. max* cv. JD17 and *G. soja* WSD nodules. Download Table S3, XLSX file, 18.3 MB. Copyright © 2021 Cui et al. 2021 Cui et al. https://creativecommons.org/licenses/by/4.0/ This content is distributed under the terms of the Creative Commons Attribution 4.0 International license.

In *G. max* nodules, there are 326 upregulated and 291 downregulated host DEGs, and 20 up- and 40 downregulated rhizobial DEGs that are common for all couples involving *rhcV* mutants compared to SF4-*G. max* (Fig. S1 and Tables S3 and S4), implying potential T3SS-dependent common adaptation pathways. In the functional enrichment analyses, the upregulated host DEGs are enriched in chromatin structure and dynamics (18 genes), cell cycle control and mitosis (10 genes), and cytoskeleton (8 genes) (*P* < 0.05, Fisher’s exact test) while downregulated ones are overrepresented by those belonging to carbohydrate metabolism and transport (14 genes), inorganic ion transport and metabolism (10 genes), and cell wall/membrane/envelope biogenesis (7 genes). Among the common downregulated genes within *G. max* nodules induced by *rhcV* mutants (Fig. S1 and Tables S3 and S4), *Glyma.06G052000* encodes a zinc transporter homolog, ZIP (51, 52), and *znuB* encodes an essential component of the high-affinity zinc uptake system of *S. fredii* (53). The *znuB* mutant of SF4 forms many pseudonodules and few normal nodules on *G. max* cv. C08, and the inactivation of Znu together with accessory zinc uptake proteins leads to downregulation of T3SS genes in the presence of the symbiotic signal genistein (53). All of these defects can be rescued by supplying replete zinc (53). These findings imply a link between the activity of rhizobial T3SS and zinc homeostasis in symbiosis, though the underlying processes remain largely unknown. It has been reported that certain effector proteins of bacterial pathogens can target chromatin access in host cells through affecting histone-modifying enzymes (54, 55) and that the expression of many nodule-specific genes correlates with ploidy-dependent opening of the chromatin together with histone tail modifications in legume nodule cells (56). Direct interactions of pathogen T3SS effectors with host cytoskeletal arsenals (such as kinesin) and the consequent promotion of virulence have been reported (57, 58). In soybean nodules, cytoskeletal arrays differ between uninfected and infected nodule cells and between developing and mature nodule cells (59). Continued reorganization of actin cytoskeleton is associated with rhizobium release from infection threads and symbiosome development (60, 61). Notably, the key symbiotic phenotype associated with the inactivation of T3SS in incompatible *Sinorhizobium* strains is the shift from uninfected pseudonodules to well-infected nodules (Fig. 1C). Therefore, it is likely that effector proteins secreted by rhizobial T3SS can be directly or indirectly involved in regulating the above-mentioned host functions.

10.1128/mSystems.01299-20.7 TABLE S4 Basic information on bacterial expression analysis (versus SF4) within *G. max* cv. JD17 nodules and *G. soja* WSD nodules. Download Table S4, XLSX file, 12.4 MB. Copyright © 2021 Cui et al. 2021 Cui et al. https://creativecommons.org/licenses/by/4.0/ This content is distributed under the terms of the Creative Commons Attribution 4.0 International license.

### Evolutionary footprints of active pangenome members.

**FIG 2:**
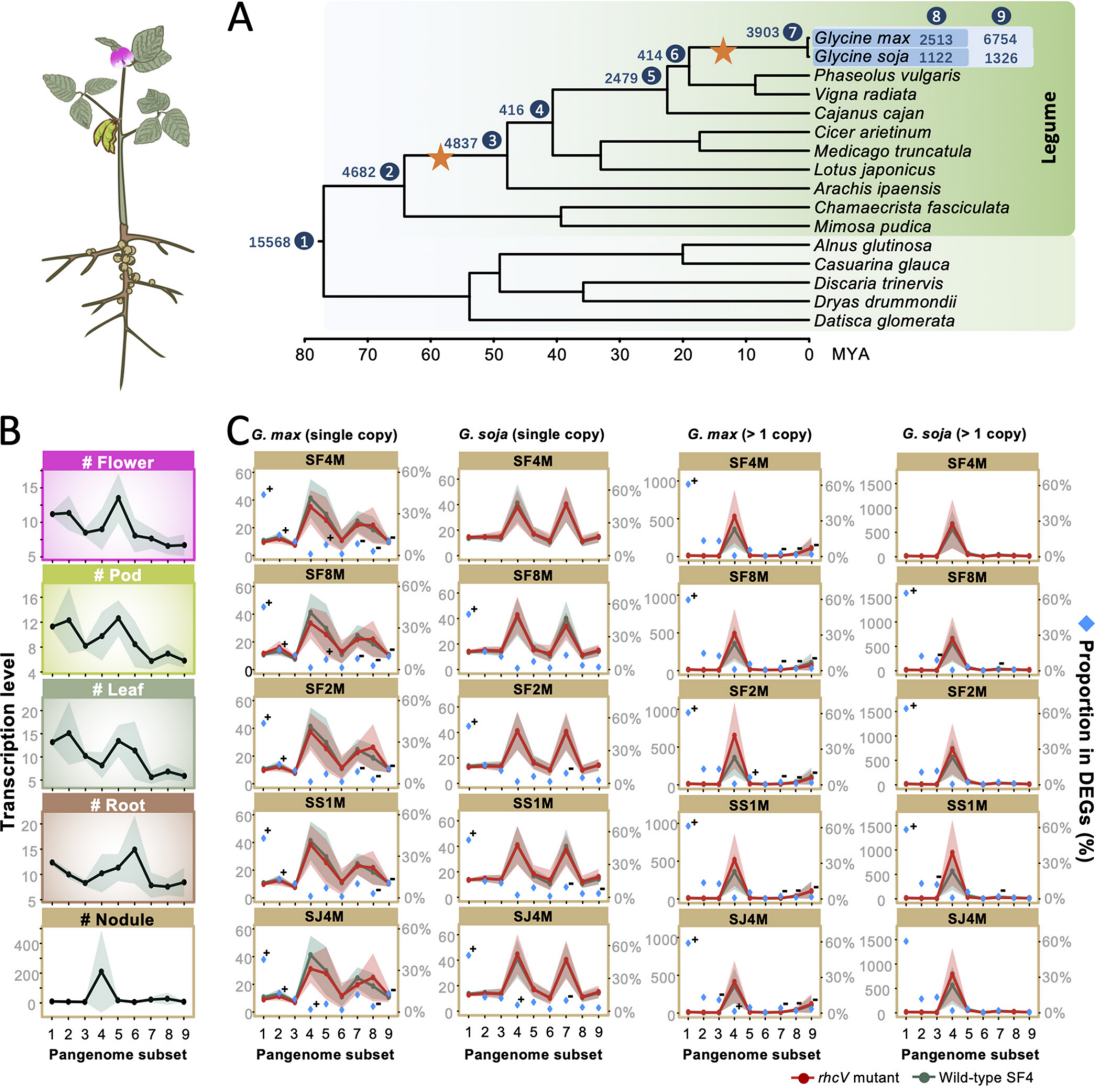
Pangenome members associated with the 58-MYA duplication event in the Papilionoids are actively transcribed in *Glycine* nodules. (A) A chronogram based on 20 single-copy genes with phylogenetic topology congruent with the phylogenomic species tree of test plants. The pangenome members of 24 Glycine max accessions and 4 *Glycine soja* accessions are divided into nine hierarchical subsets. The number of orthologous clusters for each subset is indicated. Subset 9 includes accessory genes of individual species. (B) Transcriptional levels of pangenome members in different tissues of *G. max*. (C) Transcriptional levels of single-copy genes or those with more than one copy in different pangenome subsets of *G. max* JD17 and *G. soja* WSD. Transcription levels are represented by reads per kilobase of transcript length per million reads (B) retrieved from RNA-seq data from https://soybase.org/soyseq or transcripts per million (C) obtained in this work. Proportions of DEGs belonging to individual pangenome subsets in all DEGs are shown in panel C. Confidence interval is shown for the average transcription levels in panels B and C. + and −, significant enrichment and depletion, respectively, of DEGs in individual pangenome subsets (Fisher’s exact test, alpha = 0.05).

**FIG 3:**
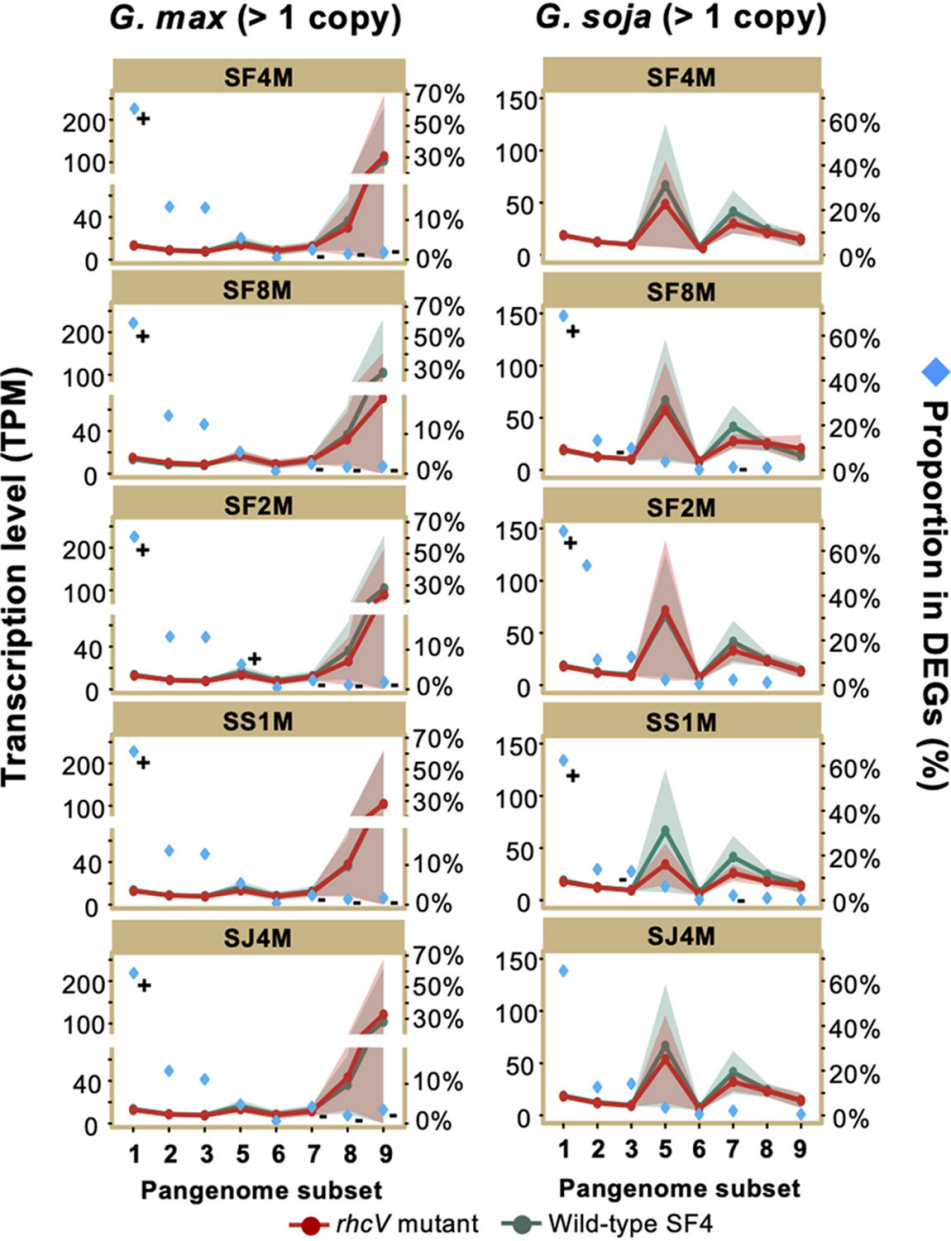
Lineage-specific pangenome members are explored by *G. max* symbiosis (left) and those associated with the 13-MYA duplication event are recruited by *G. soja* symbiosis (right). Transcriptional levels of genes with more than one copy in different pangenome subsets (except 4) of *G. max* JD17 and *G. soja* WSD. Transcription levels are represented by transcripts per million obtained. Proportions of DEGs belonging to individual pangenome subsets in all DEGs are shown. Confidence interval is shown for the average transcription levels. + and −, significant enrichment and depletion, respectively, of DEGs in individual pangenome subsets (Fisher’s exact test, alpha = 0.05).

To investigate the relative contribution to symbiosis by pangenome members evolved along the evolutionary timeline of soybeans, a chronogram (Fig. 2A) was built for *G. max*, *G. soja*, and 14 reference genomes from both legume and nonlegume species within the nitrogen-fixing root nodule clade (62, 63). Common genes of *G. max* and *G. soja* were partitioned into seven sequential subsets of different conservation levels, 1 to 7, along the nodes of the chronogram (Fig. 2A and Table S3). Moreover, accessory orthologous gene clusters shared by 24 *G. max* accessions or 4 *G. soja* accessions excluding subsets 1 to 7 were defined as subset 8, and the remaining gene clusters were defined as subset 9 (Fig. 2A and Table S3). Based on RNA-seq data from different tissues of cultivated soybean (flower, pod, leaf, root, and nodule) in SoyBase (64), the subset 4 genes are particularly active in nodules compared to the other tissues (Fig. 2B). Independent of the efficiency of test *Sinorhizobium* strains (Fig. 1C), subset 4 genes, particularly those duplicated ones, are actively recruited by both *G. max* and *G. soja* (Fig. 2C). Duplicated genes belonging to subsets 5 and 7 and single-copy ones of subsets 4 and 7 are also explored by *G. soja* symbiosis (Fig. 2C and Fig. 3). In contrast, species-specific duplicated genes belonging to subsets 8 and 9 and single-copy ones of subsets 4, 5, 7, and 8 are explored by *G. max* symbiosis (Fig. 2C and Fig. 3). An ancestral-legume genome of 7 chromosomes has been proposed (65). A whole-genome duplication event happened around 58 MYA in the Papilionoideae leading to 14 chromosomes with evidence from early-diverging Papilionoideae lineages (66), and subsequent genome rearrangements led to a putative 9 chromosomes predating the split into Hologalegina (including *Medicago*, *Cicer*, and *Lotus*) and Milletioids (including *Glycine*, *Vigna*, *Phaseolus*, and *Cajanus*) around 54 MYA (66). The subset 4 genes are shared by Hologalegina and Milletioids (Fig. 2A), and symbiotic hemoglobins with 3 to 12 copies in Hologalegina and Milletioids belong to this subset and form a monophyletic group in parallel with nonsymbiotic hemoglobins present in both legumes and nonlegumes (Fig. S3). Indeed, all symbiotic hemoglobins essential for nitrogen fixation of *G. max* nodules (67) belong to this subset. Therefore, pangenome members associated with the 58-MYA duplication event innovated key symbiotic arsenals which have been further explored by *Glycine* and other members of Milletioids and Hologalegina for maintaining nodule function. However, duplicated genes belonging to subset 7 and subsequent accessory subsets 8 and 9, at least partially associated with the 13-MYA whole-genome duplication event in *Glycine* leading to 20 chromosomes (68), are differentially explored by *G. max* and *G. soja*, implying a strong domestication effect on symbiosis of cultivated soybeans.

10.1128/mSystems.01299-20.3 FIG S3 The phylogenetic tree of phytoglobins in leguminous and nonleguminous plants forming nitrogen-fixing nodules. The protein sequences of *Glycine soja* and *Lotus japonicus* were extracted from SoyBase (□) and Kazusa DNA Research Institute (△), respectively. The protein sequences of other leguminous and nonleguminous plants were extracted from NCBI and GigaDB (*). Accession numbers are shown in brackets. Phytoglobin protein sequences were aligned with the ClustalW program, and the phylogenetic tree was constructed by the maximum-likelihood method using MEGA. Bootstrap values less than 75 are not shown. The phytoglobins of nonleguminous plants are highlighted in green. The red star indicates the putative duplication event generating symbiotic hemoglobins in the Papilionoids. Download FIG S3, TIF file, 2.5 MB. Copyright © 2021 Cui et al. 2021 Cui et al. https://creativecommons.org/licenses/by/4.0/ This content is distributed under the terms of the Creative Commons Attribution 4.0 International license.

Despite more active roles of these pangenome members after whole-genome duplication events around 58 MYA and 13 MYA in soybean nodules, DEGs are significantly enriched in subset 1 (single- and multiple-copy genes) and subset 2 (single-copy genes) in *G. max* nodules induced by individual *rhcV* mutants compared to the treatment of SF4 (Fig. 2C and Fig. 3). This suggests a considerable role of network rewiring in the more conserved subsets in symbiotic optimization. This is supported by recent phylogenomics and transcriptomics studies indicating that ancient orthologs and duplication events in plants before the existence of legume might be preadapted for symbiosis (62, 69).

**FIG 4:**
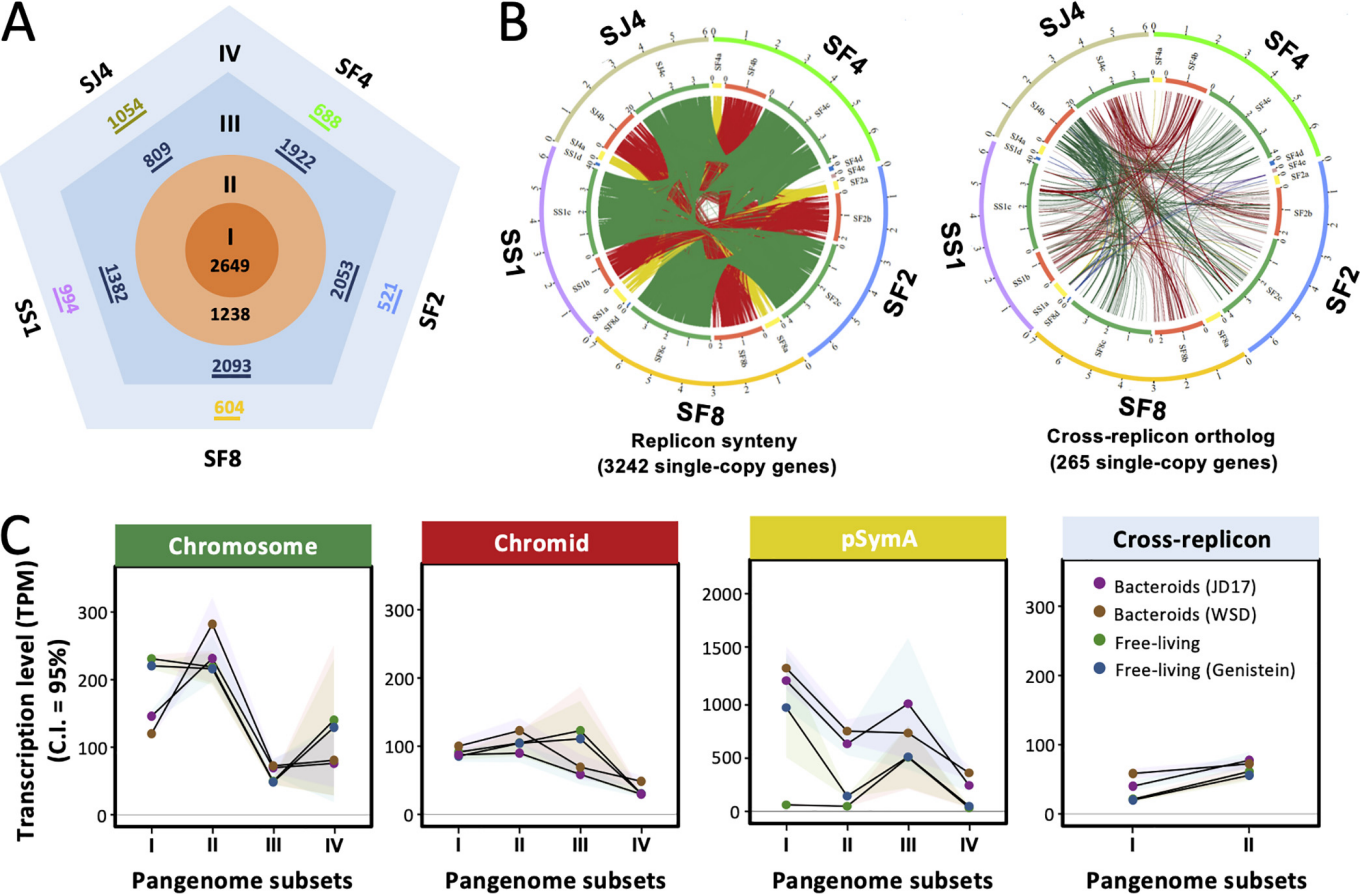
Conserved pangenome members of *Sinorhizobium* are actively transcribed in nodules. (A) Hierarchical divisions of core/accessory subsets for the five test *Sinorhizobium* strains. Genus core = subset I; core genes shared by five strains = subset I + subset II; genes shared by two to four strains = subset I + subset II + subset III; strain-specific genes = subset IV. (B) Core genes localized on the same (left) or different (right) replicons in the multipartite genomes of five test strains. Lines in green, red, and yellow represent homologs on the chromosome (SF4c, SF2c, SF8c, SS1c, and SJ4c), chromid (SF4b, SF2b, SF8b, SS1b, and SJ4b), and symbiosis plasmid (SF4a, SF2a, SF8a, SS1a, and SJ4a), respectively. Replicons are shown in a linearized way with the length of fragments corresponding to sizes in the Mb scale. (C) Replicon-dependent transcriptional levels of pangenome subsets defined in panel A. C.I., confidence interval.

To investigate contributions to nodule function by rhizobial pangenome members of different conservation levels, transcriptional levels of core and accessory genes of test *Sinorhizobium* strains were analyzed regarding different replicons. Complete genome sequences of SF4 and SF2 have been obtained in our previous work (32), and those of SF8, SS1, and SJ4 were obtained in this study (Table S6). All five strains harbor chromosome, chromid, and symbiosis plasmid (pSymA), which are conserved replicons in *Sinorhizobium* (31). Moreover, 0 to 2 accessory plasmids are present in individual strains (Table S6). By including six *Sinorhizobium* strains associated with *Medicago* (Fig. 1A), a pangenome of SF4, SF2, SF8, SS1, and SJ4 was obtained and partitioned into four subsets of different conservation levels: subset I, genus core genes (2,649); subset II, genes shared by five test strains except genus core (1,238); subset III, genes shared by two to four test strains excluding subset I and subset II (809 to 2,093); and subset IV, strain-specific genes (521 to 1,054) (Fig. 4A). There are 83.4% of core genes within subsets I and II showing a consistent location at the replicon level (Fig. 4B). These pangenome features strengthen their relationships within a monophyletic group in the phylogenomic analysis (10) and the view that *Sinorhizobium* strains isolated from either *Glycine* or *Medicago* have an open pangenome (10, 70). Twenty reference transcriptomes of SF4, SF2, SF8, SS1, and SJ4 under free-living conditions from our previous work (29) were also included here for comparison. Subsets I to IV of symbiosis plasmids (pSymA) in test strains are generally upregulated in bacteroids within nodules compared to free-living cells (Fig. 4C), and the average transcription level of a pSymA gene in nodules generally showed a positive correlation with its conservation level. This is consistent with the view that the more conserved bacterial genes are more successfully integrated in the regulation network (32, 71), while the newer pangenome members (such as those of subset IV) are more likely silenced before integration (72). Indeed, nodulation genes responding to the soybean symbiotic signal genistein (29) and those nitrogen fixation genes active in nodules belong to the more conserved subsets I and II on the symbiosis plasmid pSymA (Fig. 4C and Tables S4 and S5), and these key symbiosis genes show a low diversity in a collection of 109 *Sinorhizobium* strains isolated from soybeans in different ecoregions (73). Even though subset I genes on chromosome are generally downregulated in nodules compared to free-living cells (Fig. 4C), in line with the nongrowing status of bacteroids (21, 74), these genus core genes (subset I) and the other genes shared by test strains (subset II) are still transcribed at a high level in nodules compared to those of less conserved ones in subset III or IV (Fig. 4C).

10.1128/mSystems.01299-20.8 TABLE S5 Basic information on bacterial expression analysis (versus wild type [WT]) within *G. soja* WSD nodules. Download Table S5, XLSX file, 3.0 MB. Copyright © 2021 Cui et al. 2021 Cui et al. https://creativecommons.org/licenses/by/4.0/ This content is distributed under the terms of the Creative Commons Attribution 4.0 International license.

10.1128/mSystems.01299-20.9 TABLE S6 Overview of genome information for *Sinorhizobium* strains used in this work. Download Table S6, XLSX file, 0.01 MB. Copyright © 2021 Cui et al. 2021 Cui et al. https://creativecommons.org/licenses/by/4.0/ This content is distributed under the terms of the Creative Commons Attribution 4.0 International license.

**FIG 5:**
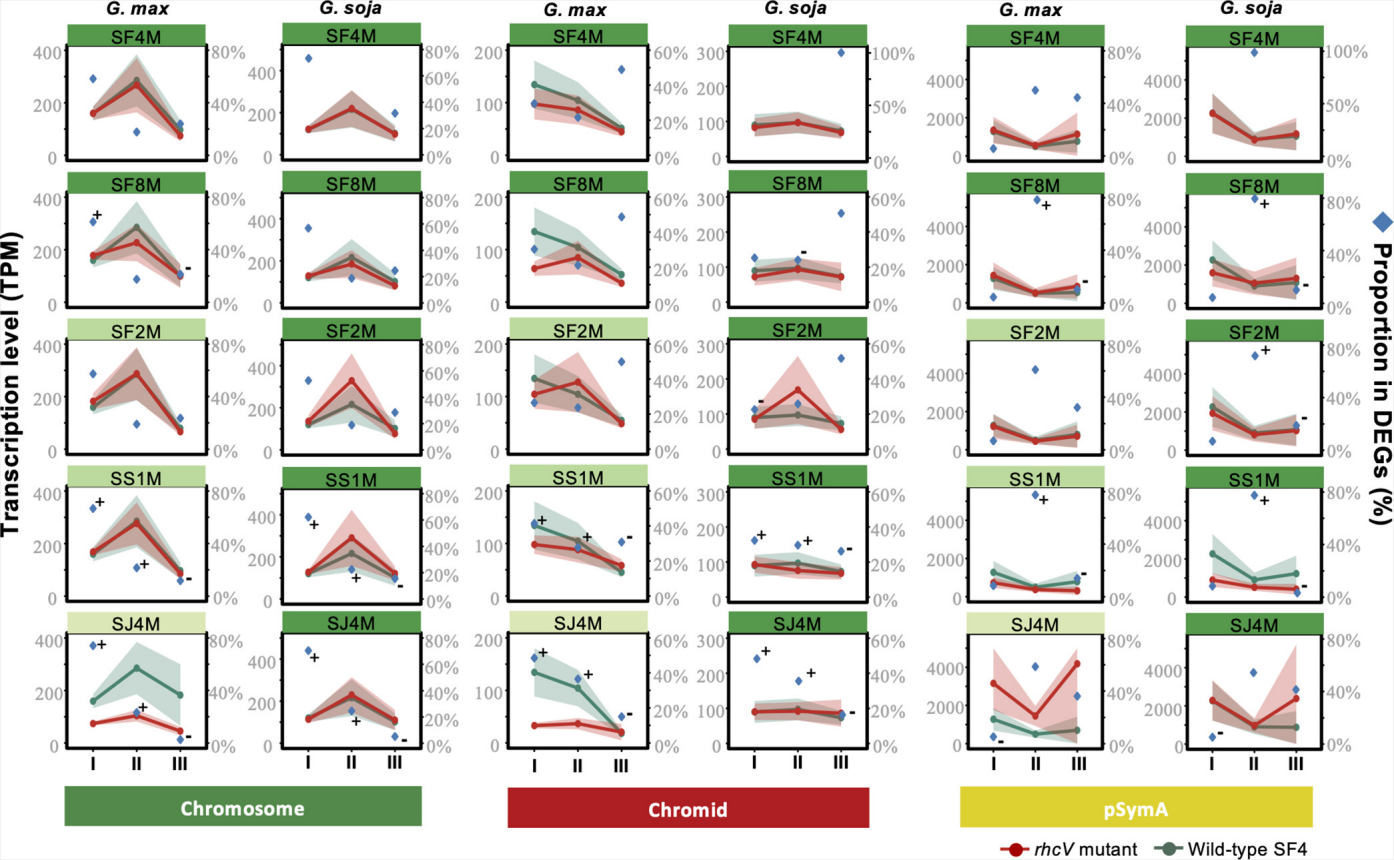
Enrichment of differentially expressed genes in conserved pangenome members predating the divergence of test *Sinorhizobium* strains. All samples from *G. max* cv. JD17 nodules (left) or *G. soja* WSD nodules (right) were compared to the treatment of SF4. Transcriptional levels are represented by transcripts per million (TPM). Proportions of DEGs belonging to individual pangenome subsets in all DEGs are shown. Confidence interval is shown for the average transcription levels. + and −, significant enrichment and depletion, respectively, of DEGs in individual pangenome subsets (Fisher’s exact test, alpha = 0.05).

DEGs are enriched in subsets I and II while depleted in subset III across chromosome and chromid in the treatment of SS1M and SJ4M compared to the treatment of SF4 in nodules of both *G. max* and *G. soja* (Fig. 5). This pattern is not observed for pSymA harboring key nodulation and nitrogen fixation genes (Fig. 5 and Tables S4 and S5). Notably, the divergence time between *Sinorhizobium* lineages associated with *Glycine* and *Medicago*, respectively, is around 106 MYA, which predates the existence of Fabaceae and the split of Papilionoideae into Milletioids (including *Glycine*) and Hologalegina (including *Medicago*) (Fig. 1A and Fig. 2A). These findings suggest a lineage-dependent network rewiring in nodules for the more conserved *Sinorhizobium* pangenome members predating the existence of legume nodulation.

### Couple-dependent symbiotic optimization by conserved pathways predating the divergence of Fabaceae or *Sinorhizobium*.

**FIG 6:**
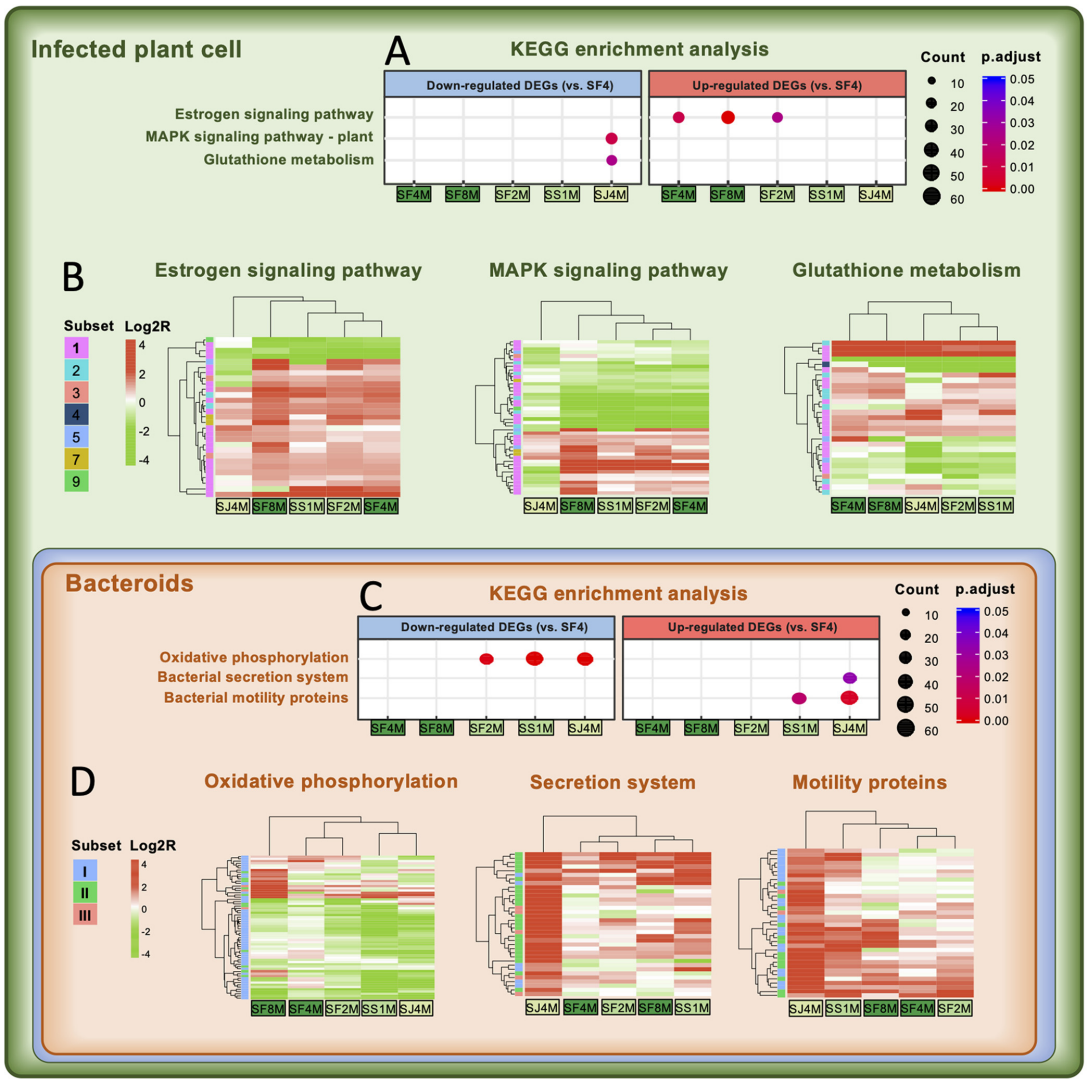
Symbiotic efficiency of Glycine max cv. JD17 nodules correlates with lineage-specific rewiring of core pathways predating the divergence of Fabaceae or *Sinorhizobium*. Function enrichment (A and C) and hierarchical clustering (B and D) analyses were performed for KEGG pathways harboring differentially expressed genes (DEGs) between treatments of individual *rhcV* mutants and that of wild-type SF4 [enrichment analysis: Fisher’s exact test, p.adjust < 0.05; hierarchical clustering analysis: red and green represent up- and downregulated genes, respectively, based on Log2R(TPM_mutant_/TPM_SF4_), and pangenome subset assignments of individual genes are indicated by different colors for both symbiotic partners].

To gain further functional insights into dual pantranscriptomes of nodules with contrasting symbiotic efficiency, KEGG enrichment analysis was performed (Fig. 6). Compared to *G. max* nodules induced by SF4, the treatments of *rhcV* mutants SF4M, SF8M, and SF2M derived from *S. fredii* strains showed a significant enrichment of upregulated host DEGs encoding various heat shock proteins and disease resistance NB-LRR (nucleotide binding site–leucine-rich repeat domain) proteins in the estrogen signaling pathway (Fig. 6A), while the low-efficiency *G. max* nodules induced by SJ4M are enriched with downregulated genes in the MAPK (mitogen-activated protein kinase) signaling pathway and glutathione metabolism (Fig. 6A). Notably, subset 1 genes are overrepresented in these three pathways (Fig. 6B), and the treatment of SJ4M exhibits the most divergent transcriptional profiles in these individual pathways. Moreover, the low- and medium-efficiency systems (SJ4M, SS1M, and SF2M) form a cluster with similar transcriptional profiles of glutathione metabolism genes.

Within the estrogen signaling pathway, two uncharacterized intracellular immune receptors (NB-LRR) are upregulated in the treatments of high-efficiency SF4M and SF8M, and Gm.14G197700, putatively involved in posttranscriptional RNA processing in NB-LRR-mediated immunity (75), is upregulated in the treatment of SF4M. The number of upregulated heat shock proteins sequentially increases in the treatment order from low- (SJ4M = 1) to medium- (SS1M = 4; SF2M = 4) to high-efficiency strains (SF4M = 7; SF8M = 11). More components at early steps of the MAPK pathway initiated by MAMP (microbe-associated molecular patterns) (76) are downregulated in the treatment of SJ4M, such as the cell surface immune receptor FLS2 recognizing bacterial flagellin (77, 78), seven RLCKs (receptor-like cytoplasmic kinases), and three MAPKs including a GmMEKK1 homolog (Gm.14G165700) negatively regulating defense responses (79). The thiol tripeptide glutathione is a well-known antioxidant and redox buffer (80), and glutathione *S*-transferase can conjugate target molecules to glutathione and act as a glutathione peroxidase to scavenge peroxides such as H_2_O_2_ (80). The number of downregulated glutathione *S*-transferases increased in the order from high- (SF4M = 2; SF8M = 3) to medium- (SF2M = 7; SS1M = 7) to low-efficiency strains (SJ4M = 10). In line with this transcriptional profile of glutathione *S*-transferase, it has been demonstrated that the downregulation of glutathione *S*-transferase in soybean nodules leads to a decrease in nitrogenase activity and an increase in oxidatively damaged proteins (81).

In bacteroids of *G. max* nodules, there is a general downshift of the oxidative phosphorylation pathway in less efficient strains (SJ4M, SS1M, and SF2M) (Fig. 6C and D and Table S4). This pathway is overrepresented by conserved subset I genes encoding respiratory components ATPase, Nuo, Cytcbb3, Cox, and Cytbd and those involved in various physiological steps producing NAD(P)H (Table S4) (23, 82). The energetically expensive processes including secretion systems T3SS and T4SS (overrepresented by subset II genes) and motility proteins (mainly subset I and II genes) are highly transcribed in SS1M and/or SJ4M (Fig. 6C). These findings indicate a low-energy status of bacteroids in these less efficient strains, though nitrogen fixation is an energetically high-cost reaction (23). In addition to these enriched pathways, notable transcriptional changes in individual conserved functional genes supporting nitrogen fixation were uncovered in low-efficiency bacteroids. The essential FeS assembly process (83) may be restricted in low-efficiency bacteroids due to the low transcription level of *sufAE*, *paaD*, and *nifU* genes (Table S4). However, the key regulator gene *nifA*; nitrogenase structural genes *nifHDK*; and *nifBENQXW*, *fdxBN*, and *fixABC* involved in FeMo-cofactor synthesis, nitrogenase maturation/stabilization, and electron donation (23, 83) are more actively transcribed in low-efficiency bacteroids than in those of SF4 (Table S4). In parallel with the disordered transcriptional profiles associated with host antioxidant functions, low-efficiency bacteroids also show a low transcription level in antioxidant functions including subset I members such as superoxide dismutase (*SJ05684_c05970*), peroxiredoxin (*SJ05684_c05210*), glutathione *S*-transferase (*SJ05684_c36640*), and thioredoxin reductase (*SJ05684_c15680* and *SJ05684_c05650*) and a subset III member peptide-methionine (*R*)-*S*-oxide reductase (*SJ05684_c06170*). These functions are involved in the dismutation of superoxide radicals, H_2_O_2_ scavenging, and various detoxification processes (82, 84, 85).

**FIG 7:**
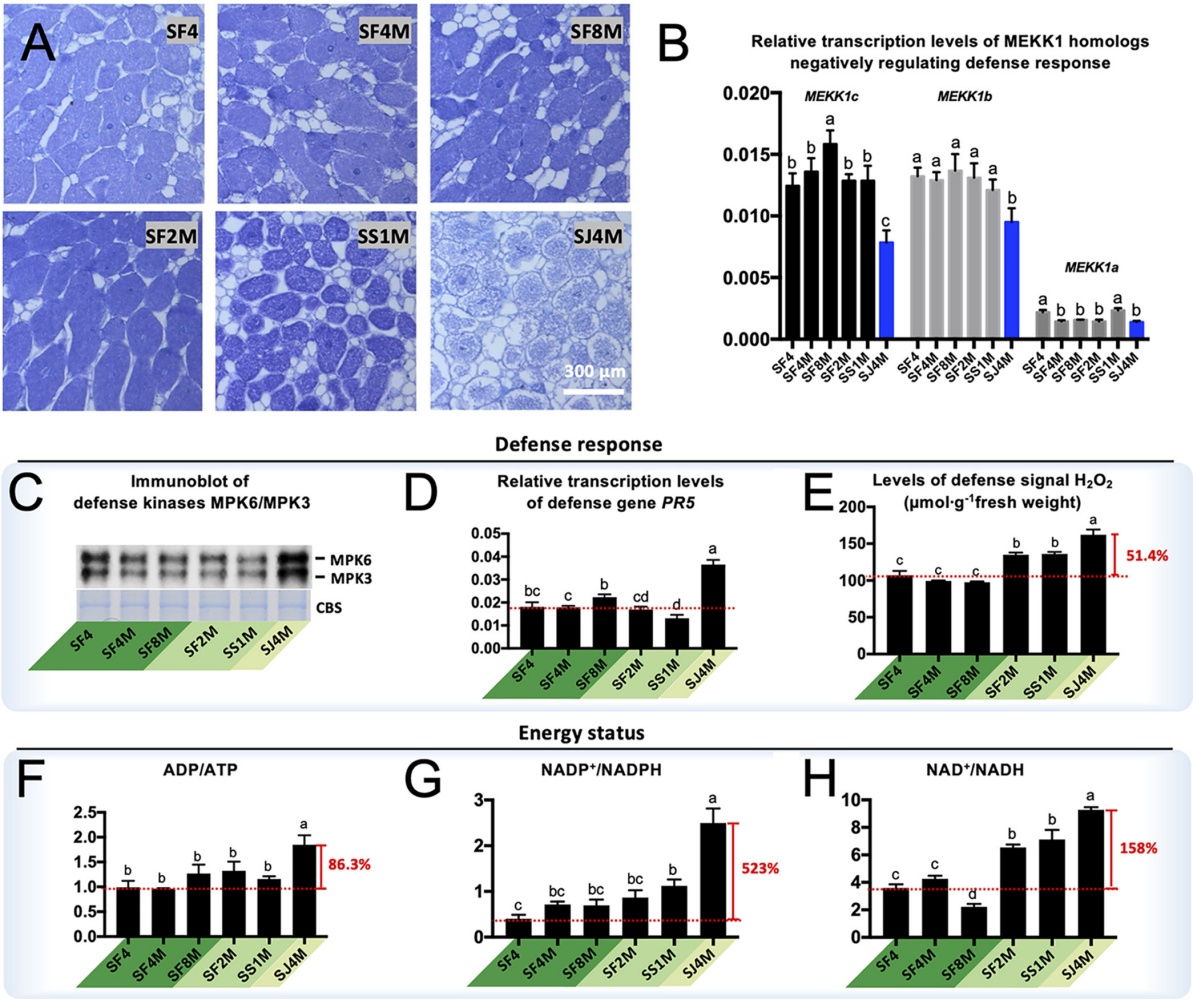
Defense response and energy status in *G. max* cv. JD17 nodules. (A) Thin sections of nodules induced by corresponding strains. (B) Relative transcription levels of MEKK1 homologs compared to an actin gene, *Gm.15G05020*. (C) Immunoblotting of defense kinases MPK6/MPK3 using antibody against phosphorylated MAPK. (D) Relative transcription levels of defense response gene *PR5* compared to *Gm.15G05020*. (E) H_2_O_2_ production. (F to H) Energy status indicated by ratios of ADP/ATP (F), NADP^+^/NADPH (G), and NAD^+^/NADH (H). Error bars represent SD from biological triplicates. Significant differences between means are indicated by different letters based on ANOVA followed by Tukey’s test (alpha = 0.05).

As described above, the couples of different symbiotic efficiencies are characterized by distinct transcriptional files of genes involved in immunity response, antioxidant functions, and energy metabolism. Indeed, *G. max* nodules infected by SJ4M have fewer bacteroids (Fig. 7A). The MAPK kinase kinase GmMEKK1 homologs negatively regulating defense responses in soybean (79) are downregulated in the SJ4M treatment (Fig. 7B, qRT-PCR). An immunoblotting experiment using these nodule samples demonstrates highly active MPK3 and MPK6 (Fig. 7C), two well-studied MAPKs which are regulated by MEKK1 and respond to oxidative stress (79, 86, 87). Among diverse defense response marker genes (88), *PR5* is transcribed at a significantly higher level in the treatment of SJ4M (Fig. 7D, qRT-PCR). All of these efficiency-related genes belong to subset 1 predating the divergence of Fabaceae.

In line with the elevated H_2_O_2_ in *GmMEKK1*-silenced soybean plants (79), the low-efficiency SJ4M treatment is characterized with the highest level of H_2_O_2_ (Fig. 7E). The medium-efficiency treatments SF2M and SS1M also exhibit a significantly higher level of H_2_O_2_ than high-efficiency SF4, SF4M, and SF8M (Fig. 7E). This phenomenon can be at least partially explained by the efficiency of rhizobial oxidative phosphorylation as measured by ratios of canonical energy currency molecules (Fig. 7F to H). As to ADP/ATP, NADP/NADPH, and NAD/NADH ratios, the low-efficiency SJ4M bacteroids are 86.3%, 523%, and 158% higher than that of SF4 bacteroids, respectively. The medium-efficiency treatments SF2M and SS1M show a significantly higher ratio of NAD/NADH than the high-efficiency treatments SF4, SF4M, and SF8M. These physiological and biochemical results provide strong evidence for the critical role of conserved pathways predating the divergence of Fabaceae and *Sinorhizobium* in optimizing symbiotic efficiency.

### Conclusion.

With conserved compositions of Nod factors (37), five related *Sinorhizobium* strains are able to induce nodules/pseudonodules on cultivated and wild soybeans. The deletion of *rhcV* or fast adaptive evolution mediated by parallel transpositions of insertion sequences into the T3SS gene cluster (29) allows bypassing standing variations in effector proteins (47) and improving compatibility to different extents among test strains. Dual pantranscriptomics uncovers that the more actively transcribed genes in nodules are those conserved symbiosis genes in rhizobia and those associated with the whole-genome duplication event in the Papilionoideae (58 MYA) in soybeans. *G. max* and *G. soja* differentially explored pangenome members of different conservation levels, particularly those associated with the whole-genome duplication event in the *Glycine* genus (13 MYA). However, the variation in symbiotic efficiency correlates with lineage-dependent rewiring of ancient core pathways predating the divergence of Fabaceae or *Sinorhizobium*, which is supported by further physiological and biochemical evidences. Collectively, innovation of key symbiotic arsenals together with the largely unexplored network rewiring in existing ancient core pathways underlines the progressive evolution of rhizobium-legume compatibility. This insight not only is significant for improving the application benefits of rhizobial inoculants in sustainable agriculture but also advances our general understanding of the interkingdom coevolution which is theoretically explored by all host-microbiota interactions.

## MATERIALS AND METHODS

### Bacterial strains, plasmids, growth condition, and soybeans.

Strains, plasmids, and primers used in this work are listed in Table S1 in the supplemental material. *Sinorhizobium* strains and Escherichia coli strains were cultured as described previously (32). To generate *rhcV* mutants, the upstream and downstream DNA fragments of target *rhcV* genes in individual genomes were amplified by PCR and cloned into pCM351, carrying a *cre-lox* system (89). The resultant pCM351 derivatives were conjugated into *Sinorhizobium* strains with the helper pRK2013, and deletion mutants were screened on the TY (tryptone yeast)-agar plates supplied with 30 μg/ml nalidixic acid and 30 μg/ml gentamicin. Candidate clones were verified by colony PCR and Sanger sequencing. To determine Rj2/Rfg1 allelic genotypes of *G. max* cv. JD17 and *G. soja* WSD, RNA samples extracted from 1-week-old seedlings by using RNAiso Plus (TaKaRa) were used to synthesize cDNA with a reverse transcription kit (Genstar). Then, two pairs of primers (Table S1) were used to amplify two fragments corresponding to polymorphic regions 452 to 490 and 731 to 758 of Rj2/Rfg1 protein (28). PCR products were subject to Sanger sequencing. The resultant sequences were compared to those of different Rj2/Rfg1 genotypes reported previously (28).

### Genome sequencing and functional annotation.

DNA of SF8, SS1, and SJ4 was extracted using the TIANamp bacterial DNA kit (Tiangen). The genomes were sequenced by PacBio and Illumina platforms and assembled at BGI-Shenzhen. Gaps were closed by Sanger sequencing of PCR products using primers listed in Table S1. Gene prediction and annotation were performed by using RAST (90) and eggNOG (91).

### Pangenomic and phylogenetic analysis.

OrthoFinder (92) with default parameters (-og) was used to identify orthogroups. Briefly, gene length bias and phylogenetic distance were removed from length-normalized similarity score, and the lower limit of orthogroup sequence similarity was defined by using RBNHs (Reciprocal Best length-Normalized hit). Genes present in all *Sinorhizobium* strains were defined as subset I. Genes shared by 5 soybean microsymbionts excluding subset I were defined as subset II, and genes shared by 2 to 4 soybean microsymbionts but not present in subset II were defined as subset III. The remaining strain-specific genes were defined as subset IV. The OrthoFinder output file Orthogroups.GeneCount.tsv contains counts of the number of genes for each strain in each orthogroup; therefore, the orthogroups that contained fewer than two genes per strain were defined as single-copy genes, but the others were defined as multiple-copy genes. Similarly to the method of gene subset definition in *Sinorhizobium*, 9 pangenome subsets of *Glycine* were defined by analyzing 4 *G. soja* and 24 *G. max* genomes available in the public database (63). The protein sequences of phytoglobins from the leguminous and nonleguminous plants were extracted from the NCBI database and aligned with the ClustalW program (93), and the phylogenetic tree was constructed by the maximum-likelihood method using MEGA (94). Single-copy genes (1,290 genes) shared by 11 *Sinorhizobium* strains and an outgroup, Rhizobium leguminosarum bv. *trifolii* WSM1689, were used to construct a maximum-likelihood phylogenetic tree by RAxML (95) with the PROTGAMMAAUTO setting using 250 bootstrap replicates. This phylogenomic tree was used as the species tree. A species tree of test plants was inferred from 25,329 orthogroups shared by all 16 plants by using the STAG (species tree inference from all genes) algorithm (96) and rooted using STRIDE (97).

### Divergence time estimation.

A Bayesian method implemented in BEAST2 (98) with an uncorrelated lognormal (UCLN) relaxed clock model was used to estimate divergence times for both bacteria and plant (parameters: Gamma Category Count = 4, Subset Model = GTR, Frequencies = Empirical). The selection of best substitution model was done with IQ-TREE (99). The Calibrated Yule Model with lognormal distribution was used for calibration with previous estimates of *Sinorhizobium-Rhizobium* divergence (203 to 346 MYA) (38) and the crown of Fabaceae (60 to 70 MYA) (41). To estimate intragenus-level divergence time of rhizobia, four high-resolution housekeeping genes (*ctaE*, *rpoB*, *rplB*, and *glpK*) (Table S7) with phylogenies congruent with the phylogenomic species tree were used. For plant, 20 single-copy core genes (Table S7) showing congruent phylogeny with the species tree were used.

10.1128/mSystems.01299-20.10 TABLE S7 Core genes used in chronogram construction. Download Table S7, XLSX file, 0.01 MB. Copyright © 2021 Cui et al. 2021 Cui et al. https://creativecommons.org/licenses/by/4.0/ This content is distributed under the terms of the Creative Commons Attribution 4.0 International license.

### Plant assays, nitrogenase activity assays, and nodule thin sections.

Seeds and rhizobial inoculants were prepared as described previously (32). Each seedling was inoculated with 1 ml rhizobial culture (optical density at 600 nm [OD_600_] = 0.2). Plants were grown at 24°C with 12-h day and night cycles for 30 days. Nitrogenase activity of nodules was measured by using the acetylene reduction method (100). Chlorophyll content was determined using a SPAD-502 meter (Konica Minolta) as described earlier (101, 102). More than three independent experiments were performed. To generate nodule thin sections, nodules were fixed in the fixative solution (90 ml 70% ethanol, 5 ml acetic acid, 5 ml 38% formaldehyde) at 4°C for 12 h. After dehydration and embedding of the nodule, 4-μm nodule slices were obtained by using a paraffin slicing machine (Leica, RM2016). Finally, nodule slices were dewaxed and stained in toluidine blue (1%) and further sealed by neutral balsam (60%).

### RNA-seq and qRT-PCR.

Nodule total RNA was extracted by using RNAiso Plus (TaKaRa). Purified RNA was quantified by using NanoPhotometer Pearl (Implen), and quality was checked by using capillary electrophoresis (Bioanalyzer; Agilent). The resultant RNA samples were subjected to mRNA enrichment, library construction, and strand-specific RNA sequencing (PE150) on an Illumina HiSeq 2000 platform following the manufacturer’s instructions (Illumina) by Novogene (The Genome Analysis Centre, Beijing, China). Two sets of nodules from two independent experiments were used.

qRT-PCR was performed by using primers listed in Table S1. In detail, 8 μg nodule or root RNA was used for reverse transcription. The StarScript II first-strand cDNA kit with genomic DNA (gDNA) remover (Genstar) was used to synthesize cDNA. qRT-PCR was performed by using 2×RealStar Green Fast Mixture (Genstar) and a Roche LightCycler480 II system. Transcription levels were normalized to the expression of the control genes, including 16S rRNA gene of bacteria and an actin gene (*Gm.15G05020*) of soybean. Triplicates from three independent experiments were used.

### Bioinformatics procedures in transcriptomic analysis.

RNA-seq reads of *Sinorhizobium* were mapped to their corresponding reference genomes with Bowtie 2 (103). RNA-seq reads from soybeans were mapped to genomes of *G. max* (Wm82.a2) and *G. soja* (PI483463.a1) obtained from SoyBase (https://soybase.org), with HISAT2 (104). Mapped reads for the protein-encoding gene in each sample were counted using featureCounts (105). Differentially expressed genes were identified by using DESeq2 (106) with an absolute fold change above 1 and adjusted *P* value of < 0.05. Hierarchical clustering was performed by using the standard R function hclust (hclust, method = average) (107). R package ClusterProfiler (108) was used in KEGG enrichment analysis. For all enrichment analyses, the annotation data set of individual genomes was used as background, and a *P* value below 0.05 indicates significant enrichment. R package ggplot2 (109) was used to visualize these enrichment results.

### Immunoblot detection of phosphorylated MPK6 and MPK3.

Nodules (4 g) were ground into powder in liquid nitrogen, and protein was extracted by 5 ml extraction buffer (50 mM Tris-MES [morpholineethanesulfonic acid], pH 8, 0.5 M sucrose, 1 mM MgCl_2_, 10 mM EDTA, 5 mM dithiothreitol [DTT], and protease inhibitor cocktail). The extract was centrifuged at 10,000 × *g* at 4°C for 30 min. Protein concentration was determined by using the Bradford method (A53225; ThermoFisher). Then, 2 μg protein was separated by SDS-PAGE (15% acrylamide gel) and transferred to nitrocellulose filter membranes by wet electrotransfer (Bio-Rad). The membrane was blocked in 1× Tris-buffered saline (TBS) buffer containing 5% nonfat milk. The blocked membrane was further incubated with anti-phospho-p44/p42 MAPK (anti-pTEpY; Cell Signaling Technology) diluted at 1:2,000 overnight at 4°C and subsequently with secondary antibody diluted at 1:5,000 for 1 h. Finally, the bands were detected using chemiluminescent horseradish peroxidase (HRP) substrate, and the image was obtained by using Tanon 5200. Three independent biological replicates were used.

### Determination of H_2_O_2_.

H_2_O_2_ concentration was determined by using hydrogen peroxide assay kit S0038 (Beyotime Biotech, China). Briefly, 2-g nodules were ground into powder in 2 ml lysis buffer S0038-3. Then, the mixture was centrifuged at 12,000 × *g* for 5 min at 4°C, and the supernatant was quickly chilled and stored at −80°C. Fifty microliters of supernatant was mixed with 100 μl test solution S0038-1 in a 96-well plate, at room temperature for 20 min, and measured immediately with a spectrometer (560 nm). Triplicates from three independent experiments were tested.

### Determination of NAD^+^/NADH, NADP^+^/NADPH, and ADP/ATP.

To enrich bacteroids from nodules, 5-g nodules were ground by using a prechilled pestle in 15 ml extraction buffer (10 mM DTT, 300 mM sucrose, 10 mM phosphate buffer, pH 7.0, 2 mM MgCl_2_, and 0.33 g polyvinylpyrrolidone [PVP]), and the mixture was centrifuged at 400 × *g* for 10 min at 4°C to remove the majority of plant cell particles. The supernatant was centrifuged at 12,000 × *g* for 10 min at 4°C. The resultant pellet was suspended with 200 μl phosphate-buffered saline (PBS) buffer (pH 7.5) and stored at −80°C. The ratios of NAD^+^/NADH and NADP^+^/NADPH were determined by using the NAD^+^/NADH assay kit (S0175) and NADP^+^/NADPH assay kit (S0179), respectively, from Beyotime Biotech, China. Briefly, 20 μl supernatant and 90 μl working solution (S0175-6 for NAD^+^/NADH and S0179-5 for NADP^+^/NADPH) were added to a 96-well plate, placed at 37°C for 20 min, and measured immediately with a spectrometer (450 nm). Cellular ADP/ATP levels were measured by EnzyLight ATP assay (BioAssay Systems, Hayward, CA). Briefly, 10 μl supernatant was mixed with 90 μl ATP reagent in a 96-well plate. After 1 min, luminescence (RLU A) was detected on a luminometer. Then 5 μl ADP reagent was added to each well and mixed by tapping the plate. After 1 min, luminescence (RLU B) was detected on a luminometer. The ADP/ATP ratio was calculated as follows: (RLUB − RLUA)/RLUA. Three independent biological replicates were used in these experiments.

### Data availability.

Raw sequence data from our RNA-seq analyses can be accessed via NCBI Sequence Read Archive (PRJNA652809). Complete genomes of five wild-type strains used in this study have been deposited in the GenBank database (BioProject no. PRJNA353922: SF8, SS1, and SJ4; BioProject no. PRJNA285929: SF4 and SF2).
