# Supplementary figures and images for: Lineage-Specific Rewiring of Core Pathways Predating Innovation of Legume Nodules Shapes Symbiotic Efficiency
Source: mSystems. 2021 Apr 13;6(2):e01299-20. doi: 10.1128/mSystems.01299-20 (PMC8547004; doi:10.1128/mSystems.01299-20)

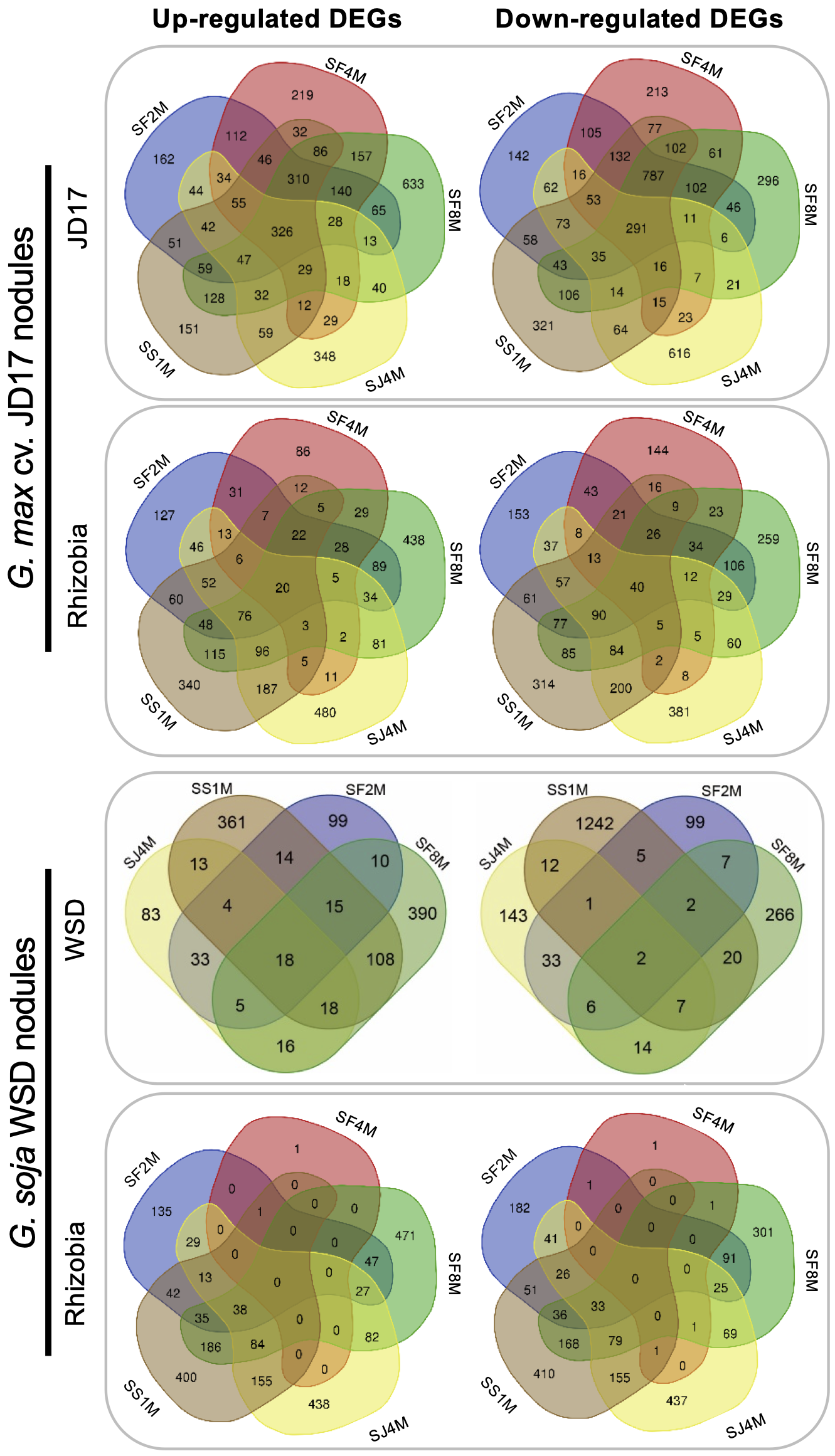

Supplement: FIG S1 [file msystems.01299-20-sf001.tif]

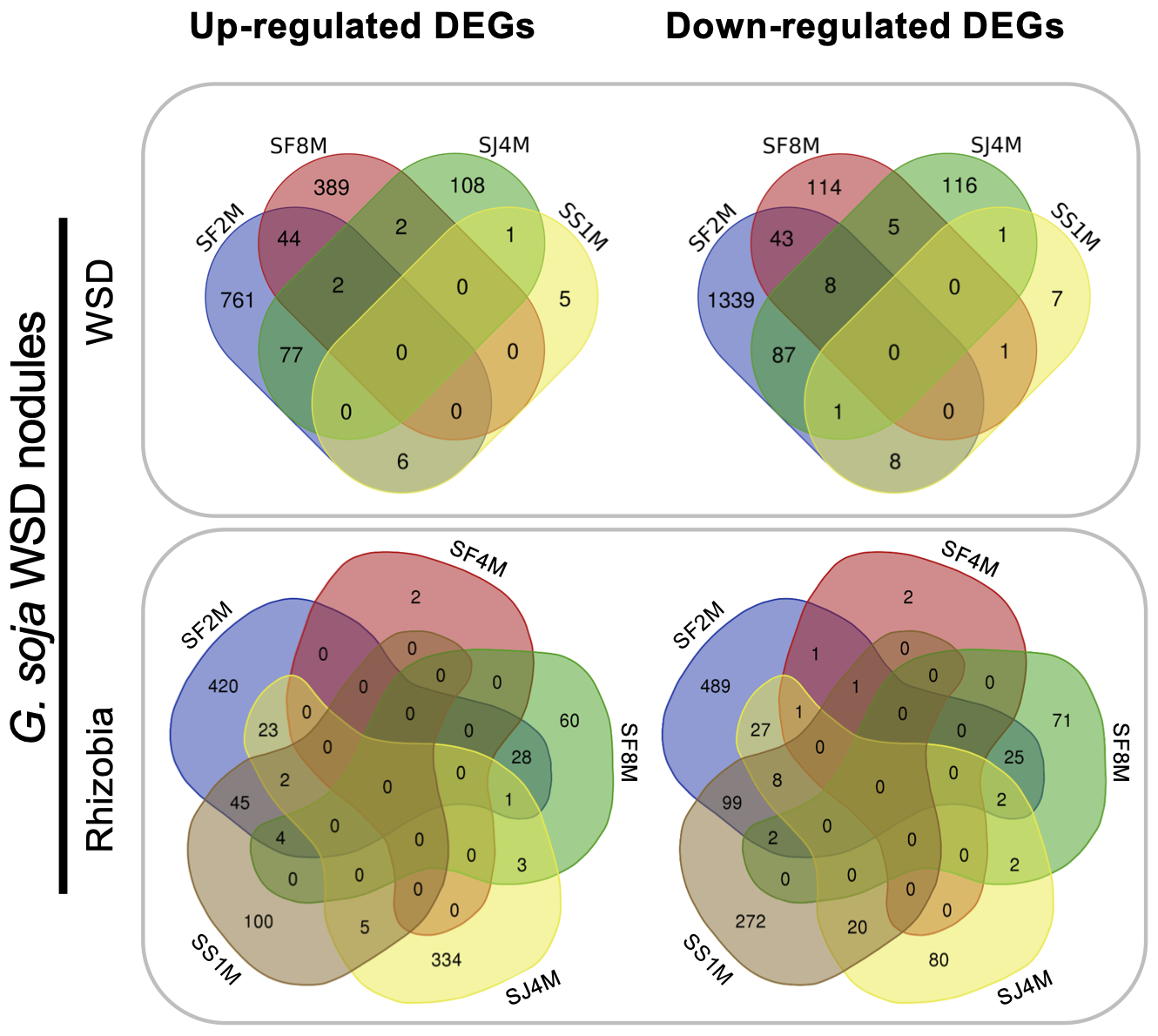

Supplement: FIG S2 [file msystems.01299-20-sf002.tif]

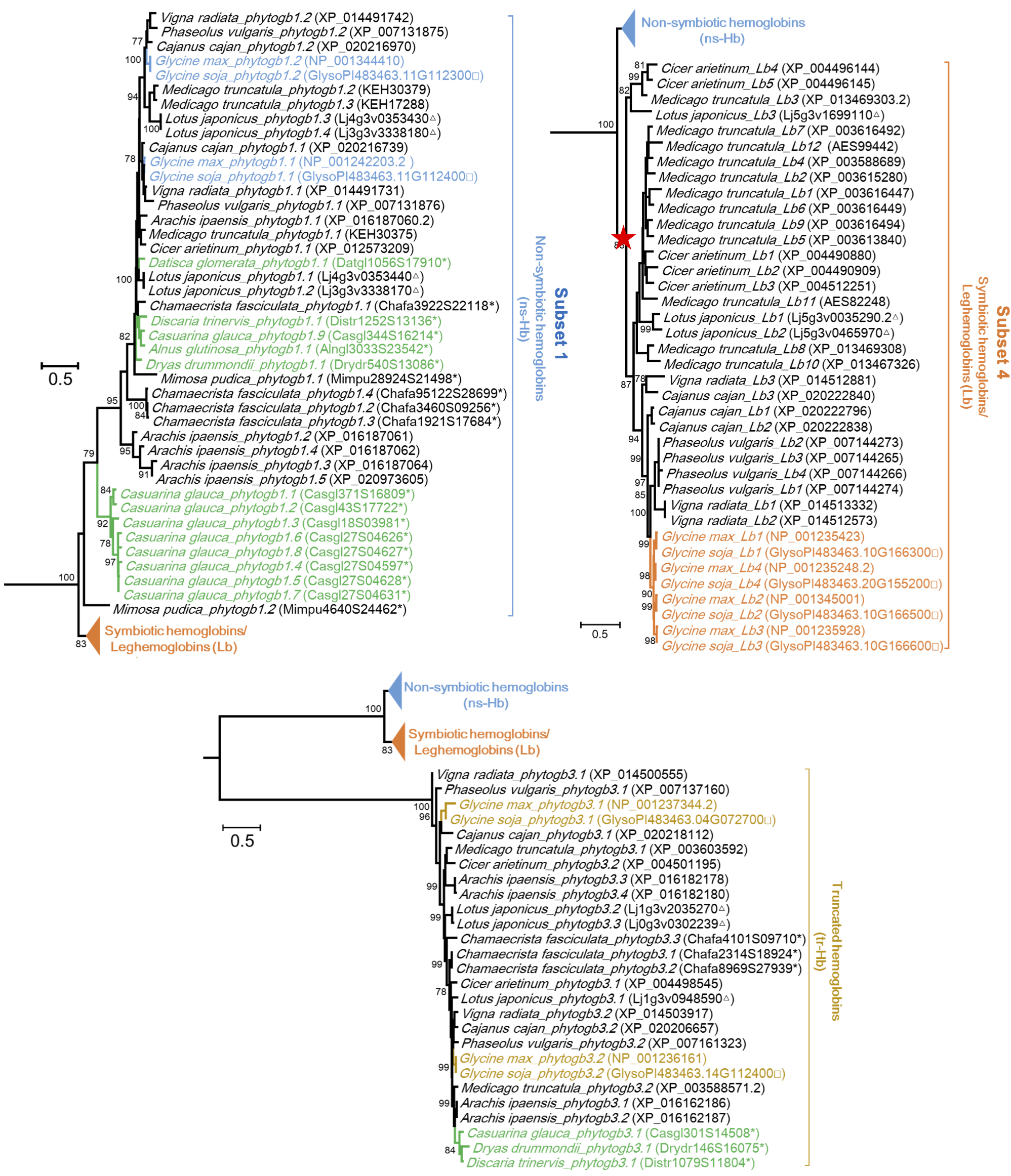

Supplement: FIG S3 [file msystems.01299-20-sf003.tif]
